# A SWOT Analysis of Portable and Low-Cost Markerless Motion Capture Systems to Assess Lower-Limb Musculoskeletal Kinematics in Sport

**DOI:** 10.3389/fspor.2021.809898

**Published:** 2022-01-25

**Authors:** Cortney Armitano-Lago, Dominic Willoughby, Adam W. Kiefer

**Affiliations:** ^1^Department of Exercise and Sport Science, University of North Carolina at Chapel Hill, Chapel Hill, NC, United States; ^2^Department of Exercise Science, Elon University, Elon, NC, United States

**Keywords:** SWOT, markerless, motion capture (Mocap), kinematics, sports medicine

## Abstract

Markerless motion capture systems are promising for the assessment of movement in more real world research and clinical settings. While the technology has come a long way in the last 20 years, it is important for researchers and clinicians to understand the capacities and considerations for implementing these types of systems. The current review provides a SWOT (Strengths, Weaknesses, Opportunities, and Threats) analysis related to the successful adoption of markerless motion capture technology for the assessment of lower-limb musculoskeletal kinematics in sport medicine and performance settings. 31 articles met the a priori inclusion criteria of this analysis. Findings from the analysis indicate that the improving accuracy of these systems *via* the refinement of machine learning algorithms, combined with their cost efficacy and the enhanced ecological validity outweighs the current weaknesses and threats. Further, the analysis makes clear that there is a need for multidisciplinary collaboration between sport scientists and computer vision scientists to develop accurate clinical and research applications that are specific to sport. While work remains to be done for broad application, markerless motion capture technology is currently on a positive trajectory and the data from this analysis provide an efficient roadmap toward widespread adoption.

## Introduction

Markerless motion capture systems have emerged as a promising tool to assess movement in both research and clinical settings. As we continue to develop and evolve the current standard of 3D motion capture, the emergence of markerless systems has provided the means to accurately measure patterns of motion in a manner that is neither invasive nor cumbersome, and that also limit the risk of measurement-induced artifacts common with marker-based systems (Mündermann et al., 2006a). Continued advancements in motion capture capabilities have supported the development of more accessible and cost-effective motion capture systems that can uniquely target a wide range of questions surrounding human movement. In the area of sports medicine and movement science, markerless motion capture offers a technological solution for evaluating unrestricted sport-specific movement patterns to better inform an athlete's risk of injury, assess rehabilitative progression, and evaluate their readiness to return to play, as well as refine skilled performance through efficient motor behavior.

One of the most exciting aspects of markerless motion capture is that can facilitate a new understanding of human movement by removing the environmental constraints of marker-based data collections (Mündermann et al., 2006a) and enable the cultivation of truly large databases of human movement (Mathis et al., 2020). In the realm of sports medicine, for example, changes in movement patterns due to injury can have a profound impact on the progression of musculoskeletal pathology, as well as the treatment of such pathologies—e.g., changes in gait mechanics following ACL injury and subsequent reconstruction have been shown to influence the progression and severity of knee osteoarthritis (Andriacchi et al., 2004; Pietrosimone et al., 2016). The ability to provide a robust kinematic assessment to address current and emerging clinical questions about factors that influence normal patterns of movement, and particularly sport-related movement, would further our understanding of human movement and provide researchers and clinicians the information necessary to enhance injury prevention, rehabilitation and general training programs.

As the accessibility of markerless motion capture software and systems seems to be markedly increasing, it is important for both researchers and clinicians to understand the up-to-date capacities of these technologies as well as areas that may require additional consideration for implementation—e.g., the computational and methodological approaches that have been taken to successfully achieve markerless motion capture. A SWOT (Strengths, Weaknesses, Opportunities, and Threats) analysis is a tool developed for strategic analysis that serves to reveal the internal strengths and weaknesses of a given entity and evaluate the external factors (opportunities and threats) the entity will face (Scholes et al., 2002). Appropriately, SWOT is an acronym for strengths, weaknesses, opportunities, and threats and is an analysis framework commonly employed to inform decision making and development. While a SWOT analysis is most commonly associated with applications in business, this framework has been applied to healthcare (Helms et al., 2008), athlete training (Düking et al., 2018), and rehabilitative technologies (Rizzo and Kim, 2005). A structured examination using a SWOT analysis of markerless motion capture systems and their application in the field of sports medicine would provide guidance and direction to the implementation of this technology.

**Figure 1:**
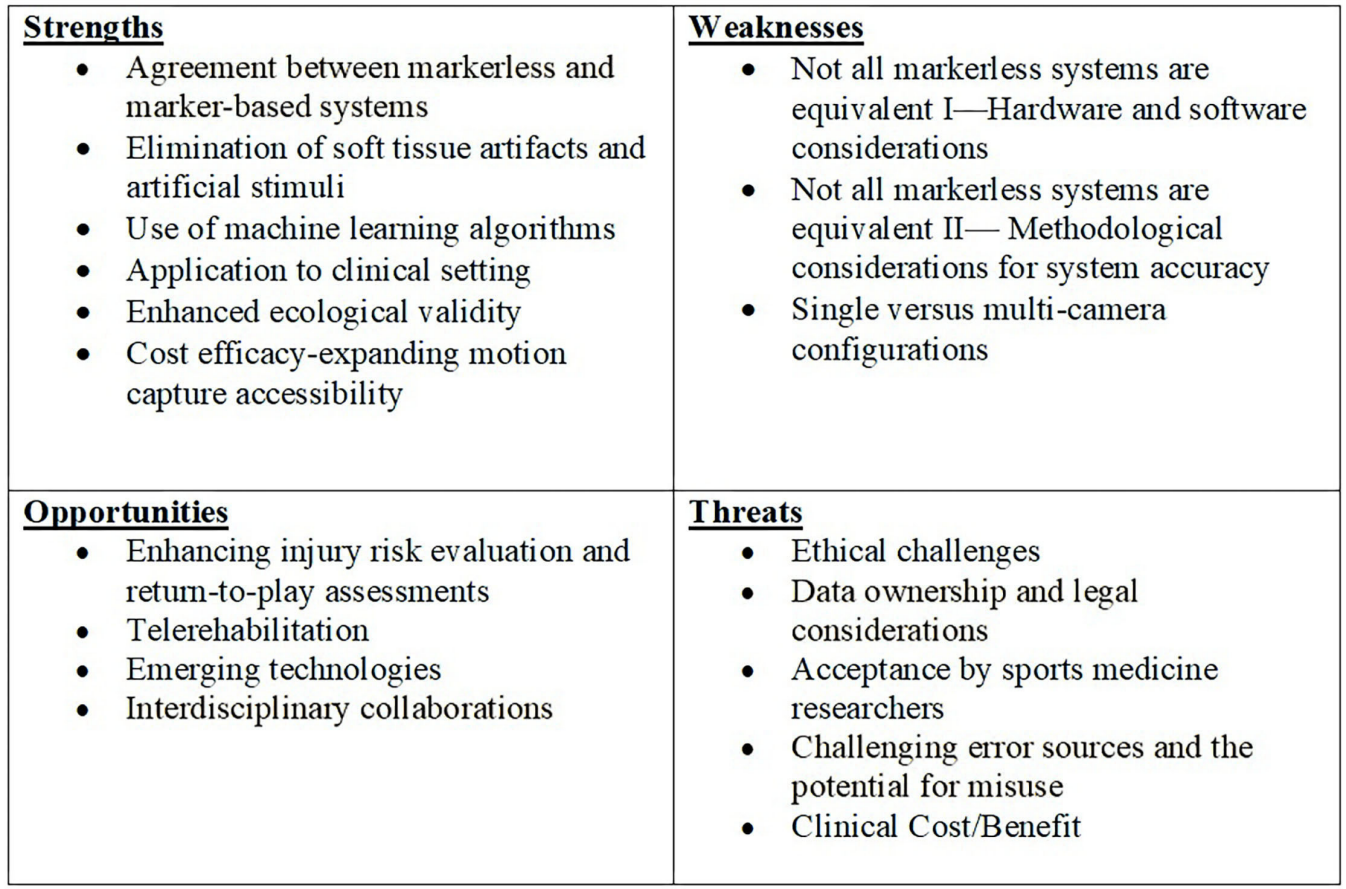
Summary of SWOT analysis for markerless motion capture.

In this SWOT analysis, the various markerless motion capture approaches used to assess lower extremity biomechanics were collectively examined to provide a broad discussion on the use and application of portable, low-cost markerless motion capture in the field of sports medicine. Ultimately, the goal of this analysis is to provide clarity on what is currently available and to identify areas for development to ensure a successful future for this technology. SWOT analyses are often criticized for their subjectivity (Pickton and Wright, 1998), and while this may be a limitation of this type of analysis, each SWOT factor has been extensively investigated through a thorough review of the current literature. To ensure a systematic review of the literature was conducted to build the foundation of the SWOT analysis, the following electronic databases were searched for relevant studies from their inception through February 2021, and a second time through December 2021: PubMed, ProQuest Health & Medical Collection, CINAHL, and Google Scholar. These electronic databases were searched using combinations of key words related to the scope of the review (search terms: lower extremity, kinematics, ankle, knee, hip, pelvis, markerless motion capture) and Boolean operators *OR* and *AND* were used to combine search terms. Of note, based on the two searches conducted, there has been a 24% increase in the research conducted within this specific scope within the last year (134 articles found during the first search in February 2021 and an additional 33 articles found in the December 2021 search), illustrating the exponential progress in this area of research. The inclusion criteria for this review were studies (a) with full- text articles available, (b) published in peer-reviewed journals, (c) in English, (d) utilizing a quantitative study design, excluding systematic reviews, (e) with human participants, (f) that evaluated the validity and/or reliability of a markerless motion capture system against a marker-based system and/or clinical assessment tool, (g) assessed lower extremity (pelvis/hip, knee, and/or ankle) kinematics, and (h) the total cost of the markerless system, cameras and software must be under $5,000.00. Thirty-one articles met all the inclusion criteria and were reviewed by the authors for the content to build and conduct the SWOT assessment (see Table 1 for study characteristics from literature review and Figure 1 for summary of SWOT analysis).

**Table 1:** Characteristics of validation studies.

**References**	**Camera(s) used for Markerless System**	**Markerless set-up**	**Validation methods**	**Task**	**Lower limb kinematics**
Capecci et al. (2016)	Kinect (v2)	Single camera	Marker-based (BTS Bioengineering System)	Squat	Knee and hip
Ceseracciu et al. (2014)	BTS Bioengineering cameras	Multi-camera	Marker-based (BTS Bioengineering System)	Walking overground	Ankle, knee, and hip
Chakraborty et al. (2020)	Kinect (v2)	Single camera	Marker-based (Optotrak System)	Walking on treadmill	Knee, hip, and pelvis
Corazza et al. (2006)	Color video cameras	Multi-camera	Virtual environment validation	Running overground	Ankle, knee, and hip
Corazza et al. (2007)	VGA cameras	Multi-camera	Marker-based	Hip abduction-adduction and flexion-extension	Joint center hip
Corazza et al. (2008)	VGA cameras	Multi-camera	Meshes from laser scan of marker-based methods	Walking overground	Ankle, knee, and hip
Corazza et al. (2010)	AVT Pike VGA color cameras	Multi-camera	Marker-based (Qualisys System)	Gymnastic movements, walking, running, and balancing tasks	Joint centers ankle, knee, and hip
Eltoukhy et al. (2017)	Kinect (v2)	Single camera	Marker-based (BTS Bioengineering System)	Star Excursion Balance Test	Ankle, knee, and hip
Gray et al. (2017)	Kinect (v2)	Single camera	Marker-based (Vicon System)	Drop vertical jump	Knee
Guess et al. (2017)	Kinect (v2)	Single camera	Marker-based (Vicon System)	Drop vertical jump and hip abduction	Knee and hip
Harsted et al. (2019)	GoPro cameras	Multi-camera	Marker-based (Vicon System)	Squat, vertical jump, box drops, drop vertical jump, and standing broad jump	Ankle, knee, and hip
Kotsifaki et al. (2018)	Kinect (v2)	Multi-camera	Marker-based (BTS Bioengineering System)	Single leg squat, single leg jump, and countermovement jump	Knee and hip
Macpherson et al. (2016)	Kinect (v1)	Single camera	Marker-based (Vicon System)	Walking and running on a treadmill	Pelvis
Mauntel et al. (2017)	Kinect (v1)	Single camera	Expert raters of the LESS	Jump landing	Knee
Mentiplay et al. (2015)	Kinect (v2)	Single camera	Marker-based (Vicon System)	Walking overground	Ankle, knee, and hip
Nakano et al. (2020)	GZRY980 video cameras	Multi-camera	Marker-based (Motion Analysis Corp)	Walking overground, countermovement jump and ball throwing	Ankle, knee, and hip
Perrott et al. (2017)	Organic motion	Multi-camera	Marker-based (Vicon System)	Knee flexion test and single limb squat	Knee
Sandau (2015)	Camera Link cameras	Multi-camera	Marker-based	Walking overground	Ankle, knee, and hip
Sandau et al. (2014)	Camera Link cameras	Multi-camera	Marker-based (Ariel Performance Analysis System)	Walking overground	Ankle, knee, and hip
Schmitz et al. (2015)	Kinect^*^	Single camera	Marker-based (Motion Analysis Corp)	Squat	Knee and hip
Tanaka et al. (2019)	Kinect (v2)	Single camera	Marker-based (Vicon System)	Functional reach test	Ankle and hip
Tipton et al. (2019)	Kinect (v2)	Single camera	Marker-based (Vicon System)	Single and double limb drop landing, Single limb hop	Knee
do Carmo Vilas-Boas et al. (2019)	Kinect (v1 and v2)	Single camera	Marker-based (Qualisys System)	Forwards and backwards walking overground	Ankle, knee, and hip
Wochatz et al. (2019)	Kinect (v2)	Single camera	Marker-based (Vicon System)	Squat, hip abduction, and lunge	Knee and hip
Xu et al. (2015)	Kinect^*^	Single camera	Marker-based (Optotrak Certus System)	Walking on a treadmill	Ankle, knee, and hip

* *Version or model not specified*.

## Strengths

### Agreement Between Markerless and Marker-Based Systems

In assessing the validity and limits of markerless motion capture, many studies have concurrently compared markerless systems to marker-based systems (Clark et al., 2013; Sandau et al., 2014; Mentiplay et al., 2015; Perrott et al., 2017; Harsted et al., 2019; Tanaka et al., 2019; Tipton et al., 2019; Wochatz et al., 2019; Drazan et al., 2021). In such studies, markerless motion capture has shown great promise. Specifically, focusing on the ability to detect lower extremity movement, multiple studies have indicated that markerless motion capture can efficiently capture spatiotemporal joint kinematic variables (Clark et al., 2013; Sandau et al., 2014; Mentiplay et al., 2015; Rocha et al., 2018) with moderate-to-high agreement during tasks such as a single leg squat (Perrott et al., 2017; Kotsifaki et al., 2018; Tipton et al., 2019), vertical jump (Drazan et al., 2021), countermovement jump (Kotsifaki et al., 2018), stair climbing (Ogawa et al., 2017), walking (Ceseracciu et al., 2014; Sandau et al., 2014; Kanko et al., 2021; Pagnon et al., 2021; Stenum et al., 2021; Takeda et al., 2021; Vafadar et al., 2021), running (Corazza et al., 2006; Macpherson et al., 2016; Pagnon et al., 2021), gymnastics tasks (Corazza et al., 2006, 2010; Mündermann et al., 2007), and clinical evaluations (Eltoukhy et al., 2017; Mauntel et al., 2021). To date, the highest accuracy with markerless motion capture has been achieved when fitting a prior articulated model to a 3D surface visual hull reconstruction using matching algorithms (Corazza et al., 2006, 2007, 2008, 2010; Mündermann et al., 2006b, 2007). More recently, however, the application of deep learning algorithms, keypoint detection approaches for biomechanical assessment are beginning to show similar or greater accuracies and illustrate significant promise for the future of markerless motion capture in the sports medicine domain (Drazan et al., 2021; Kanko et al., 2021; Needham et al., 2021; Pagnon et al., 2021; Stenum et al., 2021; Vafadar et al., 2021). It is important to note that the majority of validation studies utilizing markerless motion capture to assess joint kinematics evaluate relatively slow movements such as walking, or single plane motions such as the sagittal plane during jumping. To continue to verify the utility of these approaches for sport applications, a thorough evaluation of quicker, sport-specific movements—such as rapid change in directions, and non-linear movements—is necessary to confirm the applicability to a broad range of sports. In addition, it is important to note that with this agreement between systems, there has been some evidence to suggest that during trials using marker-based and markerless motion capture systems concurrently, the reflective markers used for tracking marker-based assessments may distort the results gleaned from cameras used for markerless motion capture (Naeemabadi et al., 2018), therefore systems compared to marker-based approaches may be better than we currently realize.

### Elimination of Marker Dependency and Environmental Restrictions

A prominent source of measurement error when using marker-based motion capture systems is skin movement artifact (Leardini et al., 2005). Soft tissue movement introduces errors of similar frequency to the actual bone movements and therefore it is difficult to parse the movement artifacts through filtering and smoothing of the data (Leardini et al., 2005). A systematic review by Peters and colleagues revealed that this artifact can be as great as 30 mm on body segments, such as the thigh, when compared to more precise methods, such as intra-cortical bone pins or X-ray radiation (Peters et al., 2010). Artificial stimuli information is also introduced with marker-based systems. Methods such as wrapping limb segments to secure clusters on the thigh or shank, the insertion of bone pins, or the attachment of numerous reflective markers introduce an artificial stimulus to the neurosensory system that can yield changes that deviate from a natural movement pattern that may mask underlying movement deficits (Mündermann et al., 2006b). The application of machine learning pose estimation algorithms offers the promise of reducing experimental error(s) either due to soft tissue movement artifact, as described above, or variability of marker placements (Szczerbik and Kalinowska, 2011), which may lead to more accurate data. Through the implementation of sophisticated pose-estimation algorithms, rather than the use of markers or clusters on the body, markerless motion capture offers a means to eliminate soft tissue artifact without requiring the use of potentially more invasive marker-based techniques (e.g., the use of intra-cortical bone pins). This affords more seamless data capture and may lead to faster and more ecologically valid data collections. In short, markerless motion capture affords the measurement of natural movement patterns that could lead to a more robust analysis of human kinematics.

### Use of Machine Learning Algorithms

One of the greatest challenges of markerless motion capture is the complexity of acquiring accurate three-dimensional kinematics without the spatial correspondence that markers deliver to marker-based systems. Fortunately, machine learning-based discriminative algorithms provide an avenue to estimate human motion. Previously captured data are used to inform a specific model (the representation of the human body) or to train the machine learning algorithm. The captured data are then input into the machine learning algorithm that is used to extract specific features from the captured image(s) to deduce explicit motions. A wide variety of machine learning algorithms have been proposed to estimate human motion (Gavrila and Davis, 1996; Bregler and Malik, 1998; Deutscher et al., 2000; Grauman et al., 2003; Baker and Kanade, 2005; Corazza et al., 2006; Moeslund et al., 2006; Poppe, 2007; Ionescu et al., 2013). Unfortunately, the research into the development and improvement of these algorithms is outside the scope for most biomechanics research and, thus, the applicability of several of these algorithms for biomechanical use is still widely unknown (Mündermann et al., 2006a; Colyer et al., 2018). That being said, more recently researchers have begun to evaluate the more popular human pose estimation algorithms (e.g., OpenPose, DeepLabCut, AlphaPose) for their accuracy when applying them to the human movement sciences domain (Drazan et al., 2021; Needham et al., 2021; Pagnon et al., 2021; Stenum et al., 2021; Takeda et al., 2021) and perhaps with the growing popularity of applying markerless motion capture to the sports medicine domain, there will be a push for more biomechanically accurate algorithms. The benefit of using machine learning-based algorithms for estimating human movement is that these algorithms can be continually refined. Specifically, data can be used to train and refine the existing algorithms to drive more accurate estimations of joint kinematics. In addition, the application of machine learning pose estimation algorithms offers the promise of reducing experimental error(s) either due to variability of marker placements (Szczerbik and Kalinowska, 2011) or skin movement artifact (see previous section *Elimination of soft tissue artifacts and artificial stimuli*) which, in turn, would result in more accurate data.

### Application to Clinical Settings

One of the goals of having a markerless system is the ease of use for compatible devices in a clinical setting to provide a valid clinical measurement tool. For a clinician, the important concern is implementation and integration into practice. If this is not supported by the technology, these systems will not be utilized. As such, researchers have investigated the ability of these systems to capture clinically relevant information for tests, such as the sit-to-stand (Otte et al., 2016), Landing Error Scoring System test (LESS; Mauntel et al., 2017, 2021), and the Star Excursion Balance Test (SEBT; Eltoukhy et al., 2017). For example, Eltoukhy et al. (2017) validated the performance of a markerless motion capture system on its ability to measure consistent results compared to manual assessment of SEBT reach results, as well as agreement with a marker-based motion capture system. The results of this study showed that the markerless system provided high agreement (ICC < 0.90) when assessing reach distance, with an error of <2 cm between systems. The conclusion was that this technology was efficient for the assessment the SEBT (Eltoukhy et al., 2017). An additional advantage of these systems for clinical assessments is their potential to provide an automated and consistent means of rapidly and accurately identifying aberrant movement patterns relevant to the clinician. For example, Mauntel et al. (2017) assessed the reliability of a markerless system used concurrently with movement assessment software in scoring the LESS as compared to scores rated by expert raters. The results showed that the markerless system was able to reliably score the LESS test and provided consistently accurate results (Mauntel et al., 2017). This finding is of great clinical relevance as it demonstrates a means toward greater throughput to clinically relevant movement assessments by (a) limiting the amount of time for the clinician to conduct such assessments (especially for an assessment such as the LESS) and (b) reducing analysis and, thus, evaluation times. These gains can also facilitate individualized interventions and enhance the quality of clinician-patient contact during treatment.

### Enhanced Ecological Validity

One of the biggest criticisms of marker-based motion capture research conducted within a laboratory setting is degree of relevance or similarity that a laboratory-based assessment has to natural movements performed on-field/on-court. While traditional laboratory- and marker-based motion capture has provided invaluable information about the characteristics of movement and movement-related deficits, the question remains whether these assessments can accurately determine if an athlete is able to return to sports without the risk of injury due to aberrant movement patterns. Research has shown that athletes can improve mechanics based on these assessments and related motion capture methods to, ultimately, reduce their risk of second injury (e.g., Paterno et al., 2010; Ardakani et al., 2019). However, the greatest predictor of a new injury is a history of previous injury (e.g., Guskiewicz et al., 2000; Salmon et al., 2005; Paterno et al., 2010; Roos et al., 2017; Losciale et al., 2019), and this indicates existing assessments and tools may not provide the most clear indication of “real world” movement function. Markerless motion capture affords the possibility to design studies to assess unrestricted movement, and to incorporate real world task contexts by assessing these movements in a sport setting. There are a number of examples illustrating the application of markerless motion capture that enhance the ecological validity by assessing movement in relevant functional environments (Parsons and Alexander, 2012; Abrams et al., 2014; Moon et al., 2019). In addition, with the continued technological advances and improved algorithms, the experimental rigor required for accurate biomechanical analysis can still be maintained while integrating the complex challenges experienced by athletes in a more natural environment (Alderson, 2015; Moon et al., 2019; Nakano et al., 2020). Thus, the data captured using markerless motion capture derived from more natural settings could have greater clinical relevance for the types of movements required of athletes on the field or court.

### Cost Efficacy—Expanding Motion Capture Accessibility

Two of the deterrents of marker-based 3D motion capture systems are the financial and time-related costs associated with such systems. Research-grade marker-based motion capture systems can cost in the tens to hundred thousands of dollars, not including the annual maintenance contracts for system hardware and software, or the employment of researcher assistants or personnel (e.g., students or technicians) who are trained to use these systems. Markerless systems provide a low-cost alternative for motion capture, providing researchers and clinicians with motion capture capabilities previously not available to them. For example, a single depth-sensing camera (often termed RGB-D camera for its capability to capture color and depth)—the latter a type of technology that comprises the Microsoft Kinect sensor (Microsoft, Redmond, WA, USA) and available on an Intel RealSense camera (Intel Corp., Santa Clara, CA, USA), for example—can be purchased for anywhere between $100 and $200. This is significantly less expensive technology that can greatly expand access to motion capture capabilities outside of the traditional laboratory setting. For example, portable markerless systems that use RGB-D cameras, or in other circumstances an off-the-shelf camera or mobile phone camera, can be deployed outside a laboratory to sport performance enhancement domains such as rehabilitation clinics or even in-home to facilitate telerehabilitation. Further, with less infringement on their personal space, patients, or participants can feel more comfortable during testing, which may lead to more diverse populations that would not have otherwise participated in motion capture research. By eliminating the preparatory time needed with marker-based systems, assessments are more time-efficient such that clinicians could feasibly utilize these technologies within their practice (Mündermann et al., 2006b), and may even be preferable in a situation with a high volume of participants [e.g., pre-season injury risk screening (Kotsifaki et al., 2018)].

### The Power of Data

One of the greatest potentials for markerless motion capture for biomechanics research lies in the potential for building data-driven tools for inferencing/predicting based on large databases (Mathis and Mathis, 2020), instead of providing observational quantitative results from relatively small samples (as seen in marker-based studies thus far). Markerless motion capture has the potential for developing truly large databases of human movement (i.e., with N in the thousands), which may lead to better statistical and predictive models of human movement (Schmidhuber, 2015; Litjens et al., 2017).

## Weaknesses

### Not All Markerless Systems Are Equivalent I—Hardware and Software Considerations

Movement analysis for clinical application requires accurate representations of joint-specific information. While markerless motion capture has been widely applied to surveillance and gaming industries, its application to the biomechanical, clinical, and sport performance enhancement domains have been limited by the accuracy of the current methods. For instance, while the studies referenced above in the “Strengths” section (*Agreement between markerless and marker-based systems* section) have reported good to high agreement between markerless and marker-based motion capture systems, the range of the error recorded within the literature suggest that markerless motion capture systems overall are still not compatible with accurate biomechanical analysis. That said, there have been some systems that have demonstrated sufficient biomechanical accuracy, especially with respect to joint positions and sagittal plane joint angles validated against marker-based systems, but this accuracy is at times plane dependent (e.g., Macpherson et al., 2016; Yeung et al., 2021).

These differences could be explained by the differences in the methods and tools used for markerless motions capture. There are different computational and methodological approaches that have been taken to successfully achieve markerless motion capture, however whether these approaches are biomechanically and clinically applicable remains an open question. There are five components that need to be considered when evaluating a markerless motion capture system: (1) the number of cameras utilized, (2) the features of the captured image(s) used, (3) the model utilized to define a human body, (4) the machine learning algorithm employed to determine the desired variables from the body model (Colyer et al., 2018), and (5) as a result of components 3 and 4, errors due to variability in anthropometrics. Given the great variety of camera configurations, model types, and algorithms that have been proposed, several variations of these markerless motion capture features can be found throughout the literature. As discussed in the *single vs. multi-camera configurations* section below, it can be expected that more cameras allows for more features to be tracked and will lead to better biomechanical results. However, this limits the generalizability of studies as it limits our ability to compare data across different markerless systems, presenting an additional challenge of clarifying the advantages of specific configurations.

### Not All Markerless Systems Are Equivalent II—Methodological Considerations for System Accuracy

There are additional methodological factors that can influence the accuracy of a markerless system that need to be taken into consideration. Specifically, four areas in particular should be highlighted. These include: (1) lighting, (2) camera range/resolution/positioning, (3) task complexity, and (4) collection environment. Current hardware limitations across available markerless systems limit their application to sports biomechanics due to a need to either (1) a need for controlled lighting conditions—e.g., markerless systems that emit light information for the acquisition and representation of human movement (Mündermann et al., 2006b), or (2) an inability to accurately capture data in direct sunlight [e.g., depth cameras such as the Kinect 1 and 2 (Zennaro et al., 2015)]. While more recent camera technologies (and algorithms) are becoming more robust to these limitations, the problem is not completely solved. An additional hardware limitation of systems such as Kinect-based markerless motion capture is that their effectiveness is only within a limited capture range (e.g., the Kinect 2 has a range of 0.5–4.5 m). Outside of the hardware limitations, several studies have noted a reduced accuracy, of the Kinect specifically, as task complexity increases (Mündermann et al., 2007; Tipton et al., 2019; Wochatz et al., 2019; Ressman et al., 2020), limiting the ability to capture and assess natural sport-specific movements of athletes. Another consideration is camera resolution—with greater resolution comes better tracking and keypoint detection. One of the weaknesses of greater resolution is the increase in the cost of the camera used as well as the processing costs. Ideally, there is a balance that needs to be met with regards to resolution and the hardware and processing costs associated. Thirdly, there is the limited generalizability of markerless motion capture models to include all body types. A challenging issue with markerless motion capture and the use of discriminative algorithms is simply if the available data is insufficient, the 2D and 3D reconstructed poses and motion trajectories will not be suitably represented. While more sophisticated models and reconstructions have been described (Corazza et al., 2010), there is a tradeoff in accuracy and processing time—increasing the accuracy also typically increases the computational burden (more time required for offline processing). Finally, one of the most attractive aspects of markerless motion capture for sports medicine is the ability to capture movement, non-invasively, during normal training environments. However, there are still environmental considerations with some markerless motion capture technology that need to be considered: (1) sunlight and (2) access to power supply and remote access such as the cloud. As previously mentioned, one consideration is sunlight. Due to its infrared properties, sunlight can introduce noise into capture devices that utilize an infrared camera (e.g., the Kinect). On a similar note, another issue would be darkness—RGB cameras are not able to track a person if it is too dark. The second environmental consideration when outside of the laboratory setting are access to a power supply for associated hardware and access to a portable hotspot in order to store data on the cloud. The latter point is going to become increasingly more important as camera resolution and subsequent data size continue to increase exponentially. As the field of sports medicine and sports science aims to leverage and validate markerless motion capture technology, these are all considerations that need to be taken into account and assessed.

### Single vs. Multi-Camera Configurations

While the most accurate markerless systems tend to have a multi-camera configuration, much literature surrounding the use of markerless motion capture for biomechanical and/or clinical assessment often only consider a single dimension *via* a single-camera setup [predominantly, the Microsoft Kinect sensor (Pfister et al., 2014; Xu et al., 2015; Eltoukhy et al., 2017; Guess et al., 2017; Mauntel et al., 2017, 2021; do Carmo Vilas-Boas et al., 2019; Tanaka et al., 2019)]. Undoubtedly, ease of use surrounding this system relative to other similar single-camera systems (e.g., the set-up, preparation, and data acquisition of these systems) is the driving factor behind researchers trying to determine its application in the sports medicine arena; however, the single camera feature might be one of the leading obstacles for these systems matching the accuracy of marker-based systems. Single-camera systems like the Microsoft Kinect were designed to capture human movement for activities performed within a limited space (capture volume of the Kinect v2 ranges from 0.5 to 4.5 m) with the human facing the device. The efficiency of these systems is thus highly dependent on camera placement relative to the subject being captured (Chakraborty et al., 2020). Accordingly, the Microsoft Kinect seems to produce comparable kinematic data [results ≤5° in the sagittal plane are assumed by this review to be clinically negligible (McGinley et al., 2009); it is important to note that not all kinematic errors are equivalent and thus the kinematic plane should be considered when evaluating accuracy] to a marker-based system when performing tasks within the optimal capture volume such as squats (Schmitz et al., 2015; Perrott et al., 2017; Mentiplay et al., 2018), or a Functional Reach Test (Tanaka et al., 2019). Movement that is outside of this optimal capture volume leads to greater difficulties for this system. Specifically, previous studies have found large differences for the estimated joint kinematics captured between a Kinect system and marker-based motion capture systems during walking (Pfister et al., 2014; Mentiplay et al., 2015; Xu et al., 2015; Guess et al., 2017; do Carmo Vilas-Boas et al., 2019), and jumping tasks (Mentiplay et al., 2018; Harsted et al., 2019; Tipton et al., 2019). Due to the complex and highly variable nature of human movement, a single camera is not properly equipped to provide sufficient 3D pose information, providing a challenge when presented with self-occlusion, or identification of another occluding object within the environment (Mündermann et al., 2006b). When compared to a single-camera system, multi-camera systems have demonstrated improved agreement and reliability in capturing the dynamic characteristics of human movement (Núñez et al., 2017; Ryselis et al., 2020). In addition, by re-identifying positions across multiple view-points, the use of multi-camera capture has shown to increase classification rates (i.e., the accuracy of the systems in identifying movements) to more than 90% (Huang et al., 2012). The robustness of markerless systems can then be increased by increasing the number of cameras. Increasing the number of cameras increases the data available to solve the given number of degrees of freedom and provide more biomechanically accurate assessments (Mündermann et al., 2006b). Algorithms are starting to more accurately identify 3D kinematics from a 2D image, but cannot replace biomechanically accurate kinematic data from markered systems.

## Opportunities

### Enhancing Injury Risk Evaluation and Return-to-Play Assessments

Computer vision-based machine learning approaches provide a powerful framework that allow for automated inferences for multiple variations of postures and movements. There is a need for the development of markerless motion capture that is easy for clinicians and biomechanists to implement and apply. The use of markerless motion capture in a natural sport environment could allow training and rehabilitation specialists on the field (such as skill coaches and athletic trainers) to determine if an athlete is at risk of injury during practice or a game situation. For example, fatigue during sport performance is associated with compensatory movement patterns believed to predispose athletes to an increased risk of injury (Shaw et al., 2008; Small et al., 2010; De Ste Croix et al., 2015; Schütte et al., 2018). The implementation of markerless motion capture could index such fatigue related risk and apply this information to individualized injury prevention and training protocols.

### Telerehabilitation

Telemedicine has emerged over the past century as a means to extend patient care and provide access to healthcare beyond a doctor's office. Broadly, the term telemedicine encompasses a wide range of telecommunications and information technologies used to facilitate the access of provider-patient (and provider-provider) health information, heath care, health education, and health care-based administrative services (Bashshur, 1995). One of the components within telemedicine is telerehabilitation—providing a range of rehabilitative services (therapeutic intervention, progression monitoring, education) to individuals without easy access to rehabilitation specialists (Theodoros et al., 2008) and can provide individualized rehabilitation outside of a hospital setting, allowing for continuous monitoring of patient progress. Research into the use of markerless motion capture systems for telerehabilitation has shown tremendous promise (Antón et al., 2013, 2016; Vukićević et al., 2015; Eichler et al., 2019; Steiner et al., 2020). To highlight a few, Antón et al. (2013) have presented a telerehabilitation system called KiReS (Kinect Rehabilitation System), a Kinect-based telerehabilitation system that allows for rehabilitation specialists to record exercises for a patient to perform and the patient can receive immediate feedback on their performance of the defined exercise (Antón et al., 2013). Vukićević et al. (2015) proposed a telerehabilitation platform based on Internet of things (IoT)—a contemporary technology aimed at improving healthcare by revamping classical methods of medical care (Jog et al., 2015)—that utilizes markerless motion capture to track and detect body movement (Vukićević et al., 2015). There is currently a gap in the literature with respect to lower limb telerehabilitation applications, with the majority of these studies only assessing posture and/or upper limb motor tasks. This may be due to the fact that current portable markerless motion capture systems seem to have better accuracy with detecting upper limb motor tasks over lower limb motor tasks (Capecci et al., 2016). In addition, despite the great potential markerless motion capture can have for telerehabilitation, the transition of telerehabilitation systems from proof-of-concept application into healthcare solutions has been challenging. One of the main challenges, highlighted by Tsiouris et al. (2020), is the lack of interoperability of these telerehabilitation platforms, beginning with the limited evidence supporting its efficacy. In order to utilize markerless motion capture to its full potential in telerehabilitation, a strong focus on developing advanced analytics for more precise outcomes and treatment plans in a user-friendly product is needed to optimize home-based rehabilitation. Markerless motion capture offers a window into addressing clinical and biomechanical challenges associated with prevention and recovery, as well as an opportunity to increase accessibility for patients to high-quality healthcare from home.

### Emerging Technologies

The recent advancements over the past decade in 3D motion capture technology and the availability of low-cost devices that afford these capabilities (e.g., Microsoft Kinect) has made the collection of 3D data more feasible than ever. This increase in 3D data has encouraged researchers to take advantage of this richer content and address several computer vision problems. For instance, the eventual hope of markerless motion capture is to have real-time 3D reconstructions of the captured data for clinical application. One of the largest obstructions technologically to real-time 3D reconstruction using markerless motion capture is local processing power capabilities. While the processing power of a computer is able to handle single camera 2D reconstructions without issue, tracking movement from multiple video camera streams is a significant challenge in the computer vision domain. The processing power bottle neck can be an issue with multicamera solutions and high-FPS single camera requirements. In both instances, solving the pose estimation may take place at lower frame rates than needed, especially when capturing movements at >90 Hz. There are a couple of strong trends, however, toward technological developments that improve markerless motion capture performance with either local or remote processing power.

The first is the push toward improving onboard processing with computer graphics processing units (GPUs). Historically, the primary drive for GPU development has been for videogame applications or animation and graphical rendering (Luebke, 2008; McClanahan, 2010). This makes GPUs ideal for computer vision applications (Greengard, 2016) and this has helped drive relevant enhancements in the markerless motion capture space (e.g., the development of CUDA by NVIDIA in 2007; Sanders and Kandrot, 2010). This has been accelerated by a number of secondary market factors, and the rapid evolution of local GPU performance, including high-end mobile GPUs, will continue to allow researchers to exploit the possibilities of 3D motion capture technologies: improving 3D object classification, 3D object recognition, and 3D shape retrieval (Ioannidou et al., 2017) on a local device.

Another alternative is the push for the use of cloud computing to run computer vision algorithms when limitations in local processing power do not allow for real-time 3D reconstructions. Cloud computing development over the past decade has gained considerable attention and has provided a platform for massive data processing and data-intensive computing (Lin et al., 2013). The fundamental concept of cloud computing is that computing takes place in the “cloud;” i.e., referring to a network of services accessed over the internet rather than from the local infrastructure of one system. This reduces the need of purchasing the physical infrastructure while providing access to data storage, computing resources, and processing capabilities that would be otherwise unattainable even with the latest in current physical technology. Efficient cloud computing requires the availability of high bandwidth network communication through which the cloud architecture provides services for large data storage and large-scale data processing. This is an important step for markerless motion capture given the computational burden required for a processing system to detect, recognize, track, and retrieve 3D data for real-time processing. Companies such as Microsoft (Microsoft Azure), Amazon (Amazon Web Services, or AWS), and Google (Google Cloud Computing Services) all provide cloud services that emphasize machine learning with strong video-based analysis options. An important benefit of these services are the reduced storage costs for longer-term (aka cold) storage (i.e., once videos are processed, they can be stored long-term if they are only accessed occasionally), as these large scale platforms have a greater capability to subsidize storage costs than local or university-wide servers. Alternatively, one of the current downsides of these cloud platforms is the potential for ongoing subscription-level service costs associated with processing time. In addition, these platforms are less user-friendly to set up and manage for those not familiar with this technological infrastructure. However, these companies are working quickly to democratize these platforms and reduce such barriers. Overall, cloud computing platforms provide an avenue to improve 3D data processing by allowing for more rapid processing with less expensive devices, while also facilitating efficient deployment outside research and development spaces and to the end-user.

### Interdisciplinary Collaborations

The driving force behind the development of markerless motion capture originates from computer vision and machine learning fields for character animation, virtual reality, smart surveillance, and the identification, recognition and tracking of human motion (Wang et al., 2003). That is to say, pose estimation algorithms were not built around biomechanical analysis or sports in general. As demonstrated by this manuscript, researchers and clinicians have expressed the value of such an application to sport or clinical settings; however, the original applications tried to fit computer-vision-based human motion analysis to non-optimized settings. Thus, the earlier pose estimation systems generally do not have the fidelity necessary for the resolution of motion capture tracking required for accurate and clinically valid biomechanical analysis. This has provided and continues to provide a unique opportunity for biomechanists and rehabilitation scientists to partner with the field of computer vision to enhance the current pose estimation solutions to increase the fidelity for the types of motions that are most pertinent to sports medicine and performance. Collaborations with computer vision specialists are critical for the development of biomechanically accurate algorithms as these professionals would have the expertise to understand the various pose estimation algorithms and models that could allow for refinement in accuracy and optimizing the relevant performance capabilities of these systems. This, in turn, would afford greater accessibility of these systems to clinicians and researchers and allow them to focus on their areas of expertise without concern for algorithmic and hardware-specific details of the technology itself. Additionally, these interdisciplinary partnerships are crucial and will continue to be very important as users of markerless motion capture begin to face new challenges, such as the organization and storage of very large video databases, building efficient database structures for human movement data, processing and reprocessing large amounts of data, and storing video data as protected health data. Such an interdisciplinary partnership would make these solutions more applicable, and may help catalyze meaningful development in this space.

## Threats

### Ethical Challenges

The feasibility of assessing athletes' movements during a sporting event or practice has radically advanced in the last decade, and it is expected to continue to evolve for the foreseeable future. Along with these technological advances in the sports medicine domain come ethical considerations that are critical to the use of technology and the end-users. As the adoption of this technology increases, scientists and health care providers will face many challenges related to information privacy and confidentiality. Specifically, the ethical dilemma becomes the protection and confidentiality of the information that can be attained through the 2D video that enables markerless motion capture. Researchers and clinicians must be equipped with the appropriate safeguards to protect and maintain the storage of video recordings, and the transmission of images and other patient record information to avoid privacy violations. It is paramount for the considerations to be discussed as this technology evolves to ensure that the personal data collected using markerless motion capture must be protected from misuse and HIPPA violations. Importantly, a few of the cloud services discussed in the previous section are well-equipped to handle these issues through the implementation of face-filter blurring, and data security practices that meet HIPPA and, in some cases, national security level approvals. Regardless, this is an important consideration for those who wish to adopt and implement this technology.

### Data Ownership and Legal Considerations

Along a similar thread as the foreseeable ethical challenges mentioned above, as these systems become more widely used in sports settings, the legal considerations surrounding ownership of the data obtained from markerless motion capture technology should be examined. In the United States, while a few states have state regulations regarding biometric privacy [e.g., California: California Consumer Privacy Act (CCPA) and the California Privacy Rights Act (CPRA); New York: Stop Hacks and Improve Electronic Data Security (SHIELD) Act; Illinois: Biometric Information Privacy Act (BIPA)], there is no comprehensive federal law regulating the collection and use of biometric data. The European Union and the United Kingdom have done a better job with the regulations they have in place (for more detail, see Tikkinen-Piri et al., 2018), however, as markerless motion capture becomes feasible and applied in sports, the issue of data ownership will be a challenging hurdle. Because this information holds considerable interest to teams and stakeholders, this is a particularly relevant threat to collegiate and professional athletes. The collection of such data raises unprecedented concerns surrounding confidentiality and data privacy, raising important questions such as who owns the data, who has access to that data, and how will this information impact an athlete's career (Karkazis and Fishman, 2017).

### Acceptance by Sports Medicine Researchers

From a researcher perspective the cost of these systems can prove quite economical, but the current computational burden and expense for biomechanically accurate markerless motion capture may be a deterrent for biomechanists. The priority for technological progression at this moment is in the honing of algorithmic techniques for markerless motion capture to enhance pose estimation accuracy at a resolution that enables the detection of subtle variations required by many biomechanical applications. This requires buy-in from the field of computer vision and sports medicine to see the potential of this application and begin those collaborations mentioned in the “*Opportunities*” section. Markerless motion capture is the future for human movement analysis; however, the speed at which we get there is strongly dependent on interprofessional collaboration and investment into the research and development that integrates biomechanical accuracy and pose estimation algorithms.

### Challenging Error Sources and the Potential for Misuse

One of the advancements that come with markerless motion capture systems is the elimination of soft tissue artifacts and errors due to marker placement found when using marker-based systems (Mündermann et al., 2006b). However, the caveat is that measurement errors in markerless data are more challenging to detect, discern, and study than those from marker-based systems. The accuracy of the machine learning algorithms is decisively determined by the choice of the underlying model as to its accuracy to functional movement (Begon et al., 2018). This includes biases that arise from training datasets, and other unknown biases due to the “black box” nature of machine learning algorithms (Mathis et al., 2020). Additionally, with the reduced cost and increased accessibility of markerless motion capture, it allows biomechanical data to be obtained, used, and interpreted by users with insufficient technical backgrounds. The lack of an adequate understanding, skill, and experience in biomechanics could propagate to errors in the reporting of kinematics and kinetics potentially leading to biomechanical data and findings that lack proper scientific rigor.

### Clinical Cost/Benefit

While the research findings of markerless motion capture offers several benefits for adoption into rehabilitative and preventative programs, these systems first must prove their value to rehabilitation specialists for adoption into everyday practice. Specifically, cost- and time-effective movement assessments are ideal so that clinicians can quickly and accurately identify movements that place an individual at a greater risk of injury or that impede recovery progress. While several existing camera and camera-like devices provide a cost-effective component of markerless motion capture, the concern comes with time efficiency of these devices in the set-up, acquisition and dissemination of movement information. The development of applications that allow clinicians to use markerless motion capture for specific movement assessments would prove quite beneficial and encourage the initial adoption of these systems. For instance, Mauntel et al. (2017) applied markerless motion capture technology to automate scoring of the LESS, a tool used to identify individuals at risk of lower extremity injury. Such an application was observed to reliably assess the LESS as expert raters and reduce the time requirements of a clinician conducting this assessment (Mauntel et al., 2017). However, the paucity of studies similar to this one limits the current understanding of clinical costs and benefits and, at present, negatively impacts mainstream markerless motion capture adoption.

## Limitations

The purpose of this SWOT analysis was to provide clarity surrounding the currently available markerless motion capture approaches and identify specific areas for future development for this technology with regards to lower extremity biomechanical assessments in sports. However, this review is not without limitations that should be considered. First, as was previously mentioned, SWOT analyses are often criticized for their subjectivity (Pickton and Wright, 1998). However, the SWOT-analysis was developed as a tool for strategic analysis, as such, each factor within this review has been thoroughly reviewed by each author. A second consideration lies within the current subject matter—markerless motion capture and its related technologies is an active area of research that progresses rapidly, thus devices and their implementation techniques can quickly become outdated. In a similar thread, there are markerless systems that have been developed that have yet been evaluated for biomechanical accuracy (e.g., the Intel3D athlete system). Therefore, while this review is based on the current technologies to date, this is an important consideration. Finally, the discussion of this review is limited to those included within the inclusion criteria framework implemented. There may be additional markerless systems available or in development that were missed based on the scope of the current review.

## Summary

Markerless motion capture systems show considerable promise for enhancing our understanding of human movement, and specifically providing unrestricted movement assessments in natural sport contexts. The emergent theme from this SWOT analysis is that despite nearly 20 years of development and discussion, markerless motion capture is still in its development stage for full application to the field of sports medicine. The success of several variants of system configurations and the encouraging initial results as well as clinical applications serve as the foundation for the future of biomechanically accurate markerless motion capture. Certain limitations still exist regarding accuracy, however these do not threaten the viability of this technology when considering the opportunities that this technology provides in the long run. The existing threats are not catastrophic as they are addressable and serve to provide valuable insight as markerless motion capture continues to develop. With thoughtful system design grounded in multidisciplinary collaborations, markerless motion capture will develop accurate clinical and research applications to expand current motion capture capabilities as well as its reach. The trajectory of this technology is positive and the future remains bright.

## Author Contributions

CA-L and AK contributed to the writing and editing of the current manuscript. CA-L led the review. DW contributed to the review of the literature and the writing of the manuscript. All authors contributed to the article and approved the submitted version.

## Funding

This work was supported, in part by an award (No. 1R21 EB027865 to AK) from the National Institute for Biomedical Imaging and Bioengineering.

## Conflict of Interest

The authors declare that the research was conducted in the absence of any commercial or financial relationships that could be construed as a potential conflict of interest.

## Publisher's Note

All claims expressed in this article are solely those of the authors and do not necessarily represent those of their affiliated organizations, or those of the publisher, the editors and the reviewers. Any product that may be evaluated in this article, or claim that may be made by its manufacturer, is not guaranteed or endorsed by the publisher.
