# Supplementary material for: A SWOT Analysis of Portable and Low-Cost Markerless Motion Capture Systems to Assess Lower-Limb Musculoskeletal Kinematics in Sport
Source: Front Sports Act Living. 2022 Jan 25;3:809898. doi: 10.3389/fspor.2021.809898 (PMC8821890; doi:10.3389/fspor.2021.809898)
Supplement: Supplementary file 1 [file Table_1.DOCX]

**Table 1. Characteristics of validation studies**

| **Author** | **Camera(s) used for Markerless System** | **Markerless Set-up** | **Validation Methods** | **Task** | **Lower Limb Kinematics** | **Validation Results** | **Algorithm Classification** |
| --- | --- | --- | --- | --- | --- | --- | --- |
| Corazza et al. 2006 | Color video cameras | Multi-camera | Virtual environment validation | Running overground | Ankle, knee, and hip | RMSE ankle inversion/eversion: 5.9°, ankle plantarflexion/dorsiflexion: 9.0°, knee adduction/abduction: 3.1°, knee flexion/extension: 4.2°, hip adduction/abduction: 2.0°, hip flexion/extenion: 3.6° | Visual hull |
| Corazza et al. 2007 | VGA cameras | Multi-camera | Marker-based | Hip abduction-adduction and flexion-extension | Joint center hip | Average absolute deviation for antero-posterior: 17.7±3.2 mm, vertical: 8.3±7.9 mm, and 9.7±11.7 mm | Visual hull |
| Corazza et al. 2008 | VGA cameras | Multi-camera | Meshes from laser scan of marker-based methods | Walking overground | Ankle, knee, and hip | Mean absolute error (generalization errors) for the ankle: 9±7 mm, knee: 14±7 mm, hip: 47±35 mm | Visual hull |
| Corazza et al. 2010 | AVT Pike VGA color cameras | Multi-camera | Marker-based (Qualisys System) | Gymnastic movements, walking, running, and balancing tasks | Joint centers ankle, knee, and hip | Mean absolute error for the ankle: 18±6 mm, knee: 14±7 mm, hip: 16±7 mm | Visual hull |
| Ceseracciu et al. 2014 | BTS Bioengineering cameras | Multi-camera | Marker-based (BTS Bioengineering System) | Walking overground | Ankle, knee, and hip | RMSD anke: dorsi/plantraflexion = 7.2°±1.8°, inversion/eversion = 7.0°±3.6°, internal/external rotation = 12.9°±7.0°; knee: flexion/extension = 11.8°±2.5°; hip: flexion/extension = 17.6°±8.5°, abduction/adduction = 14.1°±2.3°, internal/external rotation = 21.6°±9.3° | Visual hull |
| Sandau et al. 2014 | Camera Link cameras | Multi-camera | Marker-based (Ariel Performance Analysis System) | Walking overground | Ankle, knee, and hip | RMSD anke: dorsi/plantraflexion = 2.5°, inversion/eversion = 3.6°, valgus/varus = 4.3°; knee: flexion/extension = 3.5°, abduction/adduction = 2.3°, internal/external rotation = 4.8°; hip: flexion/extension =2.6°, abduction/adduction = 1.8°, internal/external rotation = 4.9° | Visual hull |
| Mentiplay et al. 2015 | Kinect (v2) | Single camera | Marker-based (Vicon System) | Walking overground | Ankle, knee, and hip | SEM(%) for comfortable paced (peak knee flexion-swing: 3.4, peak knee flexion-contact: 16.2, peak knee adduction-contact: 50.0 , total ankle flexion range: 10.6, hip flexion range: 9.1) and fast paced (peak knee flexion-swing: 4.4, peak knee flexion-contact: 13.1, peak knee adduction-contact: 57.7, total ankle flexion range: 8.8, hip flexion range: 10.7) walking | Keypoint detection |
| Sandau 2015 | Camera Link cameras | Multi-camera | Marker-based | Walking overground | Ankle, knee, and hip | The mean difference between the systems were 2.8° ± 1.1° (at heel strike), 1.8° ± 1.7° (at mid-stance), and 3.6° ± 1.5° (at toe-off; *for complete results see manuscript*) | Visual hull |
| Schmitz et al. 2015 | Kinect* | Single camera | Marker-based (Motion Analysis Corp) | Squat | Knee and hip | Average absolute difference between curves: knee flexion = 1.4°, knee adduction = 4.0°, knee internal rotation = 1.1°, hip flexion = 4.3°, hip adduction = 3.6°, hip internal rotation = 0.9° | Keypoint detection |
| Xu et al. 2015 | Kinect* | Single camera | Marker-based (Optotrak Certus System) | Walking on a treadmill | Ankle, knee, and hip | RMSE at 0.85m/s: knee flexion = 27.9±10, hip flexion = 11.8±8.6; 1.07m/s: knee flexion = 28.6±10.8, hip flexion = 11.7±8.6; 1.30m/s: knee flexion = 29±10.3, hip flexion = 11.9±8.9 | Keypoint detection |
| Capecci et al. 2016 | Kinect (v2) | Single camera | Marker-based (BTS Bioengineering System) | Squat | Knee and hip | Knee flexion RE=24.3%, AE=24±10.4 and RE=26.3%, AE=26±8.1 of the right and left legs respectively | Keypoint detection |
| Macpherson et al. 2016 | Kinect (v1) | Single camera | Marker-based (Vicon System) | Walking and running on a treadmill | Pelvis | Correlation coefficients ranged 0.41 to 0.80 for angular range of motion (for complete results see manuscript) | Keypoint detection |
| Eltoukhy et al. 2017 | Kinect (v2) | Single camera | Marker-based (BTS Bioengineering System) | Star Excursion Balance Test | Ankle, knee, and hip | Absolute mean difference in the anterior (hip: reach = 0.8±0.41, flexion/extension = 2.31±0.95, abduction/adduction = 2.29±1.32, internal/external rotation = 2.86±0.76; knee: flexion/extension = 2.46±1.29, abduction/adduction = 2.62±1.17; ankle: dorsi/plantar flexion = 2.87±1.37), posteromedial (hip: reach = 1.8±0.59, flexion/extension = 4.09±1.04, abduction/adduction = 3.19±1.22, internal/external rotation = 2.88±1.43; knee: flexion/extension = 3.29±1.27, abduction/adduction = 2.04±0.83; ankle: dorsi/plantar flexion = 2.45±1.18, reach = 2.07±0.38), and posterolateral (hip: flexion/extension = 4.95±1.83, abduction/adduction = 2.97±0.99, internal/external rotation = 5.50±4.71; knee: flexion/extension = 2.78±1.14, abduction/adduction = 5.74±1.79; ankle: dorsi/plantar flexion = 2.73±1.85) | Keypoint detection |
| Gray et al. 2017 | Kinect (v2) | Single camera | Marker-based (Vicon System) | Drop vertical jump | Knee | ICC at peak flexion: 0.95 and initial contact: 0.84 | Keypoint detection |
| Guess et al. 2017 | Kinect (v2) | Single camera | Marker-based (Vicon System) | Drop vertical jump and hip abduction | Knee and hip | Drop vertical jump correlation coefficients for both knees were >0.96 and for both hips were >0.97, for hip abduction correlations were 0.99. RMSE for drop vertical jump were <11° for knee flexion and <12° for hip flexion | Keypoint detection |
| Kotsifaki et al. 2017 | Kinect (v2) | Multi-camera | Marker-based (BTS Bioengineering System) | Single leg squat, single leg jump, and countermovement jump | Knee and hip | MDC averaged between limbs for the modified countermovement jump (thigh flexion/extension: 10.4°, rotation: 14.5°, abduction/adduction: 14.7°; shin flexion/extension: 8.9°, rotation: 16.3°, abduction/adduction: 11.9°; foot flexion/extension: 15.3°, rotation: 12.9°, abduction/adduction: 10.1°) and single leg squat (hip flexion: 11.1°, hip adduction: 8.2°, knee flexion: 7.3°, knee adduction: 7.8°) | Keypoint detection |
| Mauntel et al. 2017 | Kinect (v1) | Single camera | Expert raters of the LESS | Jump landing | Knee | Prevalence index = 0.65±0.31, bias index = 0.06±0.08 (for complete results see manuscript) | Keypoint detection |
| Perrott et al. 2017 | Organic motion | Multi-camera | Marker-based (Vicon System) | Knee flexion test and single limb squat | Knee | From the start of squat to the peak of squat, mean difference for ankle dorsiflexion = 0.9°±4.9°, knee flexion = 1.1°±7.8°, knee valgus = -1.4°±9.3°, knee rotation = 15.9°±11.6°, hip flexion = 3.5°±8.6°, hip adduction = -1.2°±6.9°, hip rotation = -3.2°±14.7° | Visual hull |
| Harsted et al. 2019 | GoPro cameras | Multi-camera | Marker-based (Vicon System) | Squat, vertical jump, box drops, drop vertical jump, and standing broad jump | Ankle, knee, and hip | Squat RMSE for ankle dorsiflexion: 4.3, knee flexion: 6.4, knee varus: 5.9, hip flexion: 11.4. Standing broad jump RMSE for ankle dorsiflexion: 11, knee flexion: 10.4, hip flexion: 16.9 | Visual hull |
| Tanaka et al. 2019 | Kinect (v2) | Single camera | Marker-based (Vicon System) | Functional reach test | Ankle and hip | Classification accuracy of the markerless system were 0.83 for the left ankle, and 0.83 for the right ankle | Keypoint detection |
| Tipton et al. 2019 | Kinect (v2) | Single camera | Marker-based (Vicon System) | Single and double limb drop landing, Single limb hop | Knee | ICC for single leg drop: peak knee flexion = 0.65(0.44-0.8), peak knee valgus = 0.59(0.23-0.79); double limb drop landing: peak knee flexion = 0.76(0.58-0.87), peak knee valgus = 0.72(0.39-0.86); single leg hop: peak knee flexion = 0.59(0.17-80), peak knee valgus = 0.55(0.25-0.75 | Keypoint detection |
| Vilas-Boas et al. 2019 | Kinect (v1 and v2) | Single camera | Marker-based (Qualisys System) | Forwards and backwards walking overground | Ankle, knee, and hip | MAE walking towards sensors: ankle: v1 = 27.21±5.57, v2 = 17.72±2.88; knee: v1 = 9.04±2.81, v2 = 5.22±1.71; hip: v1 = 6.19±2.07, v2 = 8.55±2.07; walking away from sensors: ankle: v1 = 33.63±5.30, v2 = 32.08±5.89; knee: v1 = 7.31±2.70, v2 = 7.12±2.85; hip: v1 = 7.34±2.54, v2 = 6.60±1.66 | Keypoint detection |
| Wochatz et al. 2019 | Kinect (v2) | Single camera | Marker-based (Vicon System) | Squat, hip abduction, and lunge | Knee and hip | During the squat, *r* ranged from 0.18-0.83; For the standing leg during the hip abduction exercise r ranged from 0.06–0.62 and for the moving leg r ranged from 0.16–0.59; For the lunge exercise r ranged from 0.01-0.83 for the front leg and from 0.15-0.80 for the back leg (see manuscript for complete results) | Keypoint detection |
| Chakraborty et al. 2020 | Kinect (v2) | Single camera | Marker-based (Optotrak System) | Walking on treadmill | Knee, hip, and pelvis | Case1: mean deviation of Kinect from reference system had a combined error around 36.25% with the RMSE among all joint angles across all trials around 4°. Case 2: mean deviation of Kinect from reference system had a combined error around 54.37% with the RMSE among all joint angles across all trials around 10° | Keypoint detection |
| Nakano et al. 2020 | GZRY980 video cameras | Multi-camera | Marker-based (Motion Analysis Corp) | Walking overground, countermovement jump and ball throwing | Ankle, knee, and hip | MAE range for walk (ankle: ML: 8.73-9.68, AP: 28.6-58.1, VT: 11.7-20.7; knee ML: 4.09-6.41, AP: 25.9-48.2, VT: 10.1-11.4), jump (ankle: ML: 6.67-9.82, AP: 9.31-11, VT: 20.6-27.9; knee ML: 6.47-7.74, AP: 8.48-18.3, VT: 14.8-20.9) | Keypoint detection |
| Drazan et al. 2021 | Video camera (Sony ICX285) | Single camera** need to double check, they mention two cameras but only evaluate one plane | Marker-based (Qualysis System) | Vertical jump | Ankle, knee, and hip | CMC > 0.991, RMSE < 3.22° averaged across all joints. Strong agreement was found at the hip (0.853 ± 0.23), knee (0.963 ± 0.471), and ankle  (0.970 ± 0.055) sagittal angles | Keypoint detection |
| Mauntel et al. 2021 | Kinect (v2) | Sigle camera | Marker-based (Vicon System) | Jump-landing | Knee and hip | Agreement existed between the systems (ICC rang=-1.52 to 0.96; ICC average=0.58) Agreement was better for sagittal- (ICC average = 0.84) than frontal- (ICC average = 0.35) plane measures. | Keypoint detection |
| Pagnon et al. 2021 | 4Mpixel cameras | Multi-camera | Marker-based dataset (Vicon System) | Walking overground | Ankle, knee, and hip | Flexion extension of the ankle: r = 0.35, MAE = 5.4°, knee: r = 0.93, MAE = 5.7°, hip: r = 0.97, MAE = 9.0° | Keypoint detection |
| Takeda et al. 2021 | Video camera (CASIO EX-F1). | Single camera | Marker-based (Motion analysis Corp) | Walking on treadmill | Ankle, knee, and hip | ICC for ankle: 0.51, knee: 0.92, hip: 0.97 | Keypoint detection |
| Vafadar et al. 2021 | RGB cameras (GoPro Hero 7) | Multi-camera | Marker-based (Vicon System) | Walking overground | Ankle, knee, and hip | ICC for hip: OpenPose and MAC3D = 0.97, OpenPose and Kinovea = 0.98, Kinovea and MAC3D = 0.96; knee: OpenPose and MAC3D = 0.92, OpenPose and Kinovea = 0.98, Kinovea and MAC3D = 0.90; ankle OpenPose and MAC3D = 0.51, OpenPose and Kinovea = 0.87, Kinovea and MAC3D = 0.57 | Keypoint detection |
| Stenum et al. 2021 | Video cameras* | Multi-camera | Marker-based dataset (Vicon System) | Walking overground | Ankle, knee, and hip | Sagittal plane MAE for ankle: 7.4°, knee: 5.6°, hip: 4.0° | Keypoint detection |

*version or model not specified

ICC = Interclass correlation coefficient

MAE= Mean absolute error
RMSE = Root mean square error

RMSD = Root mean square distance

MDC = Minimal detectable change

SEM = Standard error of the mean
